# Advances and Hotspots in Research on Verrucomicrobiota: Focus on Agroecosystems

**DOI:** 10.1007/s00248-025-02657-3

**Published:** 2025-11-22

**Authors:** Aleksandra Naziębło, Anna Pytlak, Adam Furtak, Jakub Dobrzyński

**Affiliations:** 1 Institute of Technology and Life Sciences – State Research Institute, Raszyn, Poland; 2 Institute of Agrophysics, Polish Academy of Sciences, Doświadczalna 4, Lublin, 20-290 Poland

**Keywords:** Soil bacteria, Ecological functions, Bacterial life strategies, Microbial ecology, Plant growth promotion

## Abstract

Members of the phylum Verrucomicrobiota are abundant yet relatively understudied soil bacteria that play key roles in biogeochemical cycling and plant–microbe interactions. They participate in the carbon (C) and nitrogen (N) cycles through the degradation of complex organic polymers such as cellulose, pectin, and starch – via the production of hydrolytic enzymes (e.g., cellulases, xylanases, chitinases), and through nitrogen transformations including denitrification, ammonification, and nitrogen fixation. Methanotrophic representatives (*Methylacidiphilum*, *Methylacidimicrobium*) oxidise methane under acidic or thermophilic conditions, thereby contributing to greenhouse gas mitigation. The ecological distribution and activity of Verrucomicrobiota are strongly influenced by nutrient availability, particularly of C, N, phosphorus (P), and potassium (K). Their variable responses to these elements reflect diverse life-history strategies, encompassing both copiotrophic (r-strategist) and oligotrophic (K-strategist) taxa. While *Spartobacteria* (e.g., *Ca.* Udaeobacter) are typically oligotrophic, classes such as Opitutia and Verrucomicrobiae exhibit mixed strategies. Beyond nutrient cycling, several members of the phylum function as plant growth-promoting and stress mitigating bacteria. They produce phytohormones (e.g., indole-3-acetic acid) and siderophores, increase the availability of nitrogen and solubilise phosphate. Some taxa exhibit antioxidant activity and can suppress phytopathogens such as *Fusarium oxysporum* through secondary metabolite production. These traits suggest a significant potential in soil health improvement. Overall, Verrucomicrobiota represent a functionally diverse and ecologically significant bacterial phylum whose metabolic versatility, adaptive life strategies, and plant-associated traits underscore their central role in sustainable agricultural ecosystems.

**Supplementary Information:**

The online version contains supplementary material available at 10.1007/s00248-025-02657-3.

## Introduction

**Fig. 1:**
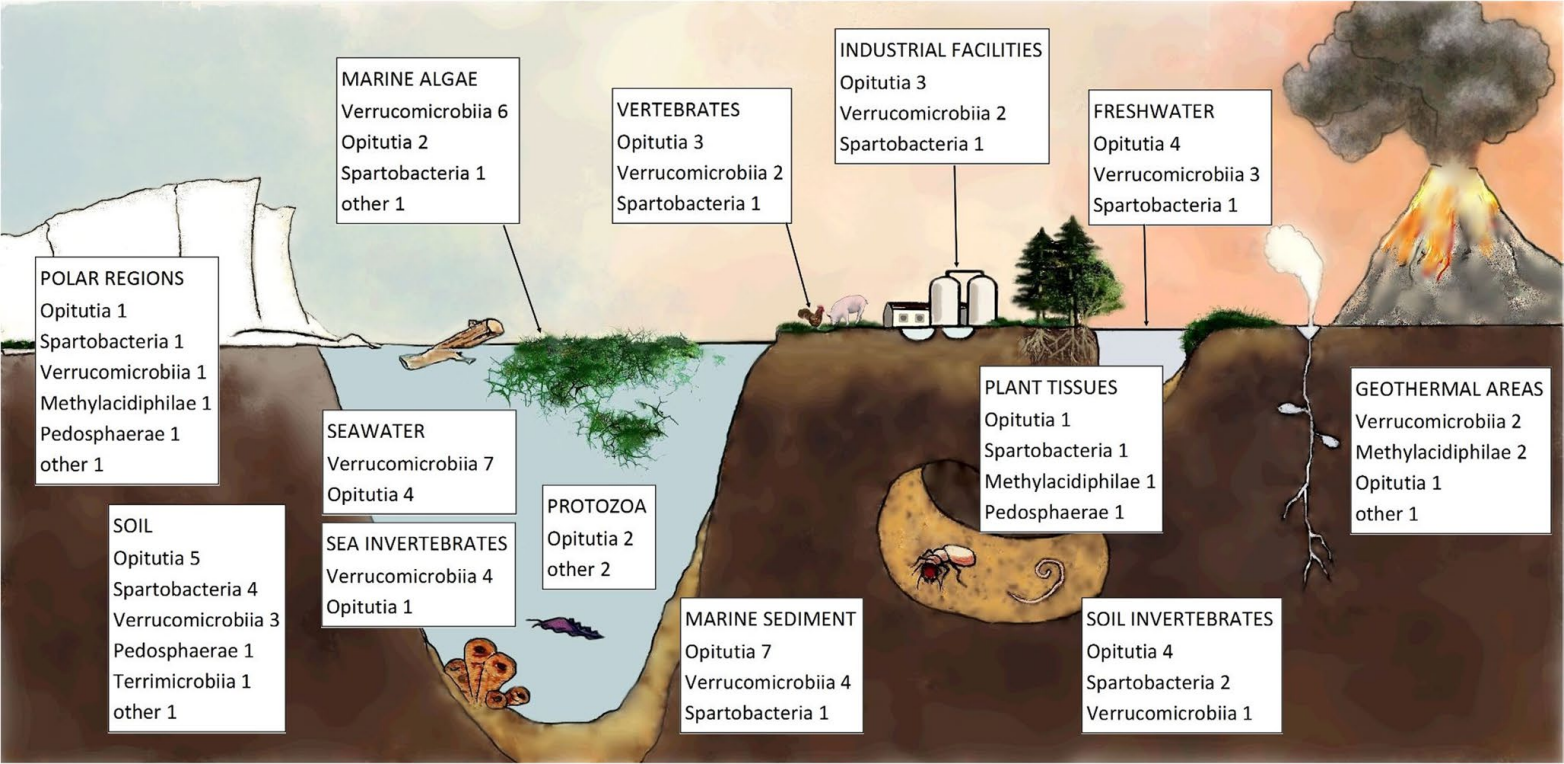
Verrucomicrobiota isolated from various environments; numbers refer to isolated genera of each class. References: [10, 12, 28, 49, 50–92]

Bacteria of the phylum Verrucomicrobiota are abundant in various environments, including freshwater sources such as drinking water, rivers, and lakes [1–3], marine environments, including marine sediments and sea organisms, particularly algae [4, 5], and anthropogenic settings such as anaerobic sludge digesters [6] and acid rock drainage [7]. They have also been found in extreme environments such as hot springs, mud volcanoes, or hypersaline lakes [8–10]. Additionally, some of them function as endosymbionts of various animals, including humans [11, 12]. Although only a few genera were found in soil (Fig. 1), the 16 S rRNA gene analyses show that members of the phylum Verrucomicrobiota may be predominant in this environment [13]. However, their relative abundance is probably underestimated, as Verrucomicrobiota are among the least explored bacterial phyla, and many representatives of this taxon are unculturable microorganisms. According to the published research, Verrucomicrobiota account for 0.3% to as much as 15% of the total bacterial community in the topsoil of arable fields and grasslands [1, 14–27]. In general, their relative abundance is higher in woodland soils than in open habitats, and rises with sampling depth [13, 28–30].

Verrucomicrobiota represent a distinct evolutionary lineage within the PVC supergroup (Planctomycetes–Verrucomicrobia–Chlamydia–Lentisphaera), and are phylogenetically closest to Chlamydiota, suggesting a common ancestor and an independent evolutionary trajectory within bacteria [93]. Over evolutionary time, members of this phylum have developed unique adaptations, including conserved signature indels (CSIs) in proteins that may serve as molecular markers and facilitate specialisation to diverse ecological niches, such as soil, freshwater, marine, and extreme environment**s** [93].

Verrucomicrobiota were first observed in the 1930 s [94, 95], but it took researchers as long as almost 40 further years to obtain the first pure culture [96]. The scarcity of culturable representatives and the shortcomings of environmental research methods of that period resulted in Verrucomicrobiota being officially recognised as a separate phylum as late as in 1997 [97]. However, even after that, the presence of Verrucomicrobiota remained under-reported [13]. One of the reasons is considered to be the mismatch of “universal” bacterial primers, commonly used for sequence-based surveys [13], as well as the resistance of the cells of this phylum to some DNA extraction methods [98].

Nowadays, the widespread application of primer sets designed to amplify with few biases against Verrucomicrobiota, such as the commonly used primers for the amplification of hypervariable regions (V1-V9) in the 16 S rRNA gene (e.g., F515/R806 targeting the V4 region), has shed new light on the environmental distribution of these microorganisms [99]. Furthermore, the continuous discovery of new bacteria and advances in phylogenetic analysis are leading to an ongoing redefinition and reorganisation of this phylum. Currently, it is divided into several classes: Methylacidiphilae, Opitutia, Pedosphaerae, Spartobacteria, Terrimicrobiia, and Verrucomicrobiia [100, 101]. The up-to-date taxonomic classification of soil Verrucomicrobiota is presented in Table 1. The best-studied genera include *Opitutus* (Opitutia class), *Chthoniobacter* (Spartobacteria class) and *Luteolibacter* (Verrucomicrobiia class), among others [31, 102, 103]. Verrucomicrobiota are found in a wide range of soil environments, including forest [104], grassland [15], and agricultural soil, e.g. paddy fields [105]. Their relative abundance depends on soil conditions, among which pH, organic matter content, and the availability of biogenic elements [26, 106, 107] are most important. In general, most Verrucomicrobiota cultured from soil are believed to be free-living [108, 109], and most are mesophilic [110], facultatively or obligately anaerobic bacteria [32].

**Table 1:** Taxonomic classification of verrucomicrobial strains associated to plants and soil environment. The names of genera in brackets originate from the publication by Bünger et al. [37] and cannot be found in databases such as NCBI and DSMZ

Class	Opitutia		Verrucomicrobiia	Pedosphaerae	Spartobacteria		Terrimicrobiia	unclassified
Order	Opitutales	Puniceicoccales	Verrucomicrobiales	Pedosphaerales	Chthoniobacterales	not assigned	Terrimicrobiales	
Genus	*Lacunisphaera*	*Coraliomargarita*	*Luteolibacter*	*Pedosphaera*	*Chthoniobacter*	*Ca.* Xiphinematobacter	*Terrimicrobium*	*Methylacidiphilum*
	*Opitutus*		*Rubritalea*		*Ca.* Udaeobacter	(*Spartobacter*)		(*Astrumicrobium*)
	*Congregicoccus*		*Prosthecobacter*					
	*Termitidicoccus*		*Roseimicrobium*					
	*Horticoccus*		*Verrucomicrobium*					
	*Geminisphaera*							
	(*Albicoccus*)							

Until recently, many studies have associated the relative abundance of Verrucomicrobiota with low contents of C and other nutrients and most members of the phylum were considered oligotrophs. Compared to copiotrophic bacteria, oligotrophs are less dependent on the availability of biogenic elements, and thrive in low-nutrient environments [33]. Oligotrophic bacteria are characterised by a slow growth rate, which was also recognised as the underlying reason for the difficulty in obtaining culturable isolates of Verrucomicrobiota [111, 112]. However, recent research based on high throughput sequencing techniques has shown reverse patterns implying that some representatives of the phylum may be copiotrophic [113].

Verrucomicrobiota are not only ubiquitous, but they are involved in a multitude of biogeochemical processes, such as carbon and nitrogen cycles [32]. For example, some representatives of the Verrucomicrobiota are capable of oxidising methane, a potent greenhouse gas. This is especially important nowadays, when increasing anthropogenic impact (also resulting from agricultural activity) threatens soil ecosystem health [114]. The reduction of atmospheric methane concentration is recognised as the fastest opportunity to curb the rate of global warming [115] and microbial processes are in the spotlight as critical tools to achieve this goal [116].

An in-depth understanding of verrucomicrobial ecophysiology is important for a proper comprehension of microbial communities’ dynamics in soil and their role in agroecosystems.

Considering the above, the purpose of this review is to:

- provide an up-to-date overview of Verrucomicrobiota, with a focus on their responses to agricultural practices and life strategies;
- verify the general assumption that this phylum predominantly comprises oligotrophic bacteria;
- reassess the ecological roles of Verrucomicrobiota and identify existing knowledge gaps.

## Review Methods

The literature survey is based on Google Scholar, Scopus, and Web of Science databases. A number of keywords and their combinations were used to search for publications – for instance “Verrucomicrobiota”, “microbial community”, “bacteria”, “soil”, etc. Articles cited in the reference lists of each publication were also examined. Finally, over 180 peer-reviewed research articles were selected (a more detailed description can be found in the Supplement). The figures were prepared using VOSviewer, Krita, Paint and MS Office.

## Bibliometric Landscape of Verrucomicrobiota - Focused Research

**Fig. 2:**
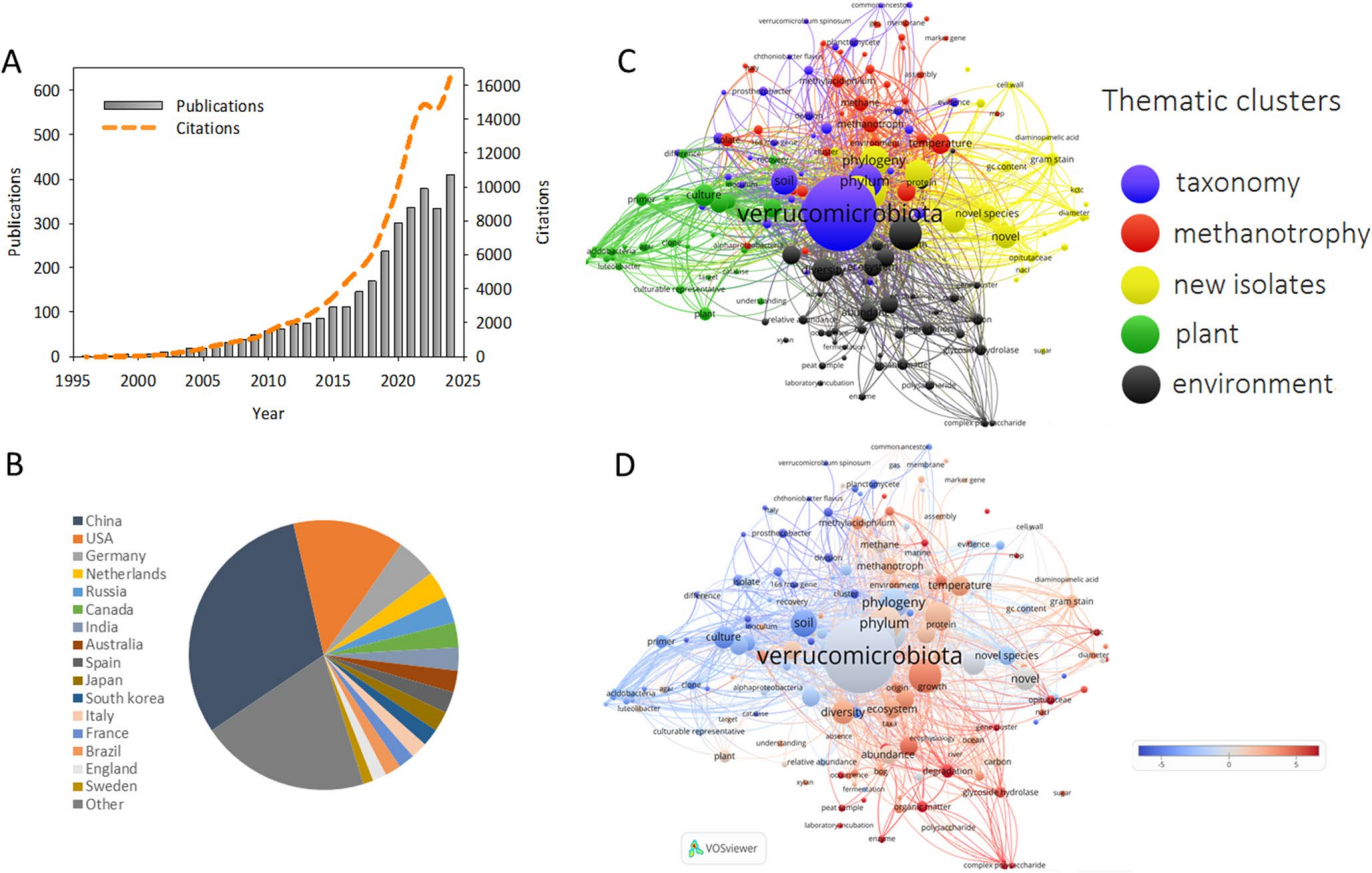
Descriptive statistical analyses of articles on Verrucomicrobiota published from 1997 to 2024. (**A**) annual publication numbers and citation times of publications, (**B**) global geographical distribution of publications, and VOSviewer based network visualisation of keyword co-occurrence: (**C**) keyword cluster map revealing major research themes, (**D**) a chronological overview of keywords based on the average publication year (scores were normalised by subtraction of the mean value), blue colour denote oldest while red – newest records)

As presented in Fig. 2a, the recent decade has witnessed a noticeable increase in both the annual publication number and the number of citations of Verrucomicrobiota - focused studies. The total number of citations of collected publications from 1997 to 2025 was 112,650, indicating that Verrucomicrobiota have attracted significant research interest over the past 10 years and are likely to be a research hotspot in 2025 and beyond (Fig. 2a). More than half of the articles came from Asian countries, with 31% of the total number originating in China, followed by the USA (13%) – Fig. 2b.

In general, the literature describing research on Verrucomicrobiota falls into 5 clusters, visualised in separate colours in Fig. 2c. Cluster 1 “environmental” (black) was the largest. It contained terms related to the research on the occurrence and role of Verrucomicrobiota in various ecosystems, their potential in the transformation of organic matter, including the ability to degrade macromolecules. Among the described environments, peatland and aquatic ecosystems are best represented. Cluster 2 “methanotrophic” (red) brings together terms referring to the unique group of acidophilic methanotrophs, their occurrence, ecophysiology, and adaptation mechanisms to extreme environments. Cluster 3 “taxonomic” (blue) consists of terms associated with the taxonomic positioning of Verrucomicrobiota. Cluster 4 “plant-related” (green) groups together terms connected with studies of the occurrence and role of Verrucomicrobiota in the plant rhizosphere and attempts to culture microorganisms belonging to this taxon. Cluster 5 (yellow) is distinguished from the others by terminology related to the isolation and description of new taxa/isolates.

As seen in the overlay visualisation Fig. 2d, the subject of research on Verrucomicrobiota has been changing over time. Earlier studies mainly dealt with aspects related to establishing their tentative taxonomic position and attempts to obtain culturable representatives. More recently, the attention of researchers has been attracted more by the role of Verrucomicrobiota in the environment and their contribution to the degradation of organic matter. Currently, there is also a high level of interest in the methanotrophic Verrucomicrobiota. The underlying reason for this scientific activity is the unique role of these microorganisms in reducing methane emissions from extreme environments (often acidophilic and thermophilic), where geological processes are the source of CH_4_ and the canonical (proteobacterial) methanotrophs are not able to survive.

Apart from methanotrophy, topics related to greenhouse gases emission or uptake have not been significantly addressed in research on Verrucomicrobiota. It can be considered an important knowledge gap, given that these microorganisms are ubiquitous and may play a significant role in the biomass decomposition of primary producers [34]. Furthermore, Verrucomicrobiota possess a metabolic apparatus capable of N_2_O transformation [34, 117] but their role in regulating emissions of this potent greenhouse gas also remains poorly recognised.

## The Role of Verrucomicrobiota in the Agroecosystems

To date, dozens of strains from genera within Verrucomicrobiota have been isolated from various environments, including *Opitutus* (Optituae class), *Chthoniobacter* (Spartobacteria class), and *Prosthecobacter* (Verrucomicrobiia class) [118, 119]. However, still little is known about soil Verrucomicrobiota, mostly due to their limited culturability. According to Hu et al. [120], unculturable bacteria are not only very abundant (about 30–56% of bacterial taxa and their metabolites), but also necessary for a complete understanding of soil ecology. Although Verrucomicrobiota have not been extensively studied, their high relative abundance indicates they play an important role in soil ecosystems. They participate in nutrient cycling and exhibit potential for biocontrol and bioremediation [121–123]. Some of them, like the genus *Pedosphaera*, have been identified as linked to soil fertility and plant health [35].

###  Carbon Cycling

According to numerous studies, members of the phylum Verrucomicrobiota are an important element of the soil C cycle. They contribute to the decomposition of the most common biopolymers, primarily structural and storage polysaccharides such as cellulose, pectin, and starch, breaking them down into simpler compounds that can be utilised by other microorganisms and plants. This is largely due to their ability to produce hydrolytic enzymes, including cellulases, xylanases, and chitinases. For example, *Opitutus terrae* and *Chthoniobacter flavus* are known to decompose complex organic compounds, facilitating mineralisation of soil C [32, 36, 118]. Bünger et al. [37] found high densities of glycoside hydrolase genes in four rice-associated isolates, including two rhizosphere and two endophytic strains. A recent study employing deep graph convolutional neural networks method has revealed a strong link of the Verrucomicrobiaceae family with C dynamics in soil [124]. Beyond soil isolates, various other bacterial strains within the Verrucomicrobiota phylum exhibit the ability to hydrolyse a wide range of polymers such as lignin, xylan, starch, galactan, galactomannan, glucomannan, xyloglucan, pullulan, arabinan, lichenan, beta-glucan, pachyman, locust bean gum, xanthan gum, fucoidan, and agar [10, 34, 38, 39].

Verrucomicrobial methanotrophs, inhabiting volcanic soils, have been relatively recently discovered [40] and are distinguished not only by the taxonomic affiliation but also by ecophysiology. Representatives of the described genera (*Methylacidiphilum*,* Methylacidimicrobium*, *Candidatus* Methylacidithermus) are mostly acidophilic, with growth optimum in the range 55–65 °C (e.g. *Methylacidiphilum infernorum* V4). However, mesophilic representatives are also known, mostly within *Methylacidimicrobium* (e.g. M. sp. LP2A) [41].

So far, only a limited number of studies have reported the presence of verrucomicrobial methanotrophs in soils outside the environments associated with volcanic activity. This, however, is expected to change with the development of research techniques. Kaupper et al. [125], who studied methanotrophic activity in ombrotrophic peatlands, confirmed the presence and active CH_4_ assimilation by *Methylacidiphilaceae*. Vaksmaa et al. [126] found aerobic verrucomicrobial methanotrophs in a rice paddy field in Italy, while Kroeger et al. [127] detected representatives of the genus *Methylacidiphilum* in amazonian pastures. Nevertheless, it should be noted that the methanotrophic activity of Verrucomicrobiota outside the extreme environments still requires verification [128].

Due to the significant role of Verrucomicrobiota in the soil carbon cycle, their relative abundance often responds to fertilisation with carbon-containing organic amendments. However, the literature reports highly variable responses, which are likely related to the diverse life-history strategies (r- or K-strategists) and functional traits (e.g., distinct enzymatic profiles) exhibited by different members of the Verrucomicrobiota in soil ecosystems (Table 2).

**Table 2:** Impact of fertilisation and soil parameters on the abundance of Verrucomicrobiota. Only taxa lower than phylum are presented in column 1. 1 – positive effect; −1 – negative effect; n.e. – no effect

Taxon	Fertilisation	C	*N*	*P*	K	pH	Plant species	Soil type	Source publication
Opitutus	NK fertilisation		n.e.		1		*Zea mays*	non-calcareous fluvo-acquic soil	[129]
Opitutus	N (urea)/cow manure	n.e.	−1	−1	n.e.	n.e.	*Camellia sinensis*	brown loamy	[22]
Opitutus	N (NH_4_NO_3_ and urea)P		n.e.				grassland (mostly *Leymus chinensis*)	saline-sodic chernozem	[130]
Opitutus	NPK	1		1	1		*Fritillaria taipanensis*	fine-loamy, mixed, mesic Aridic Haplustalf	[36]
Opitutus	chicken manure		1	1	1		*Brassica oleracea*	–	[131]
Lacunisphaera	N fertilisation (urea)	1	1				*Malus sieversii*	peat–vermiculite–apple orchard soil	[132]
Opitutaceae		n.e.	1	1		n.e.	*Zea mays*,* vegetables*	contaminated with heavy metals	[133]
Coraliomargarita	NPK + pig manure/crop straw				1		*Triticum aestivum/Oryza sativa*	paddy soil (Wushan soil)	[50]
Opitutia	biochar/bagasse organic fertiliser	1/−1					*Malus domestica*	–	[134]
Luteolibacter	NPKSCa/chicken manure + *Trichoderma guizhouense*	1		1		−1	*Lycopersicum esculentum*	saline soil	[135]
Luteolibacter	organic fertiliser	1	n.e.	n.e.		1	*Panax quinquefolius*		[136]
Luteolibacter	PKSNaMg + organic/inorganic N	1	−1				*Triticum aestivum*	–	[137]
Prosthecobacter	PKSNaMg + organic/inorganic N	1	−1				*Triticum aestivum*	–	[137]
Prosthecobacter	mineral NPK		1	1	1		*Brassica oleracea*	–	[131]
Verrucomicrobiaceae	chemical fertiliser	−1		−1	−1		*Triticum aestivum*	silt loam	[138]
Verrucomicrobiia	NPK	−1	1				*Pisum sativum/Zea mays*	Aridisol	[27]
Pedosphaera	N (urea), PK		−1	n.e.	1		*Oryza sativa*	sandy loam	[139]
Pedosphaeraceae	sheep manure + mushroom residue + *Aspergillus brunneoviolaceus*/NPK	−1	−1	−1		−1	*Brassica chinensis*	acrisols (acidic red soil)	[106]
Pedosphaerales	NPK + organic fertiliser					1	*Tectona grandis*	yellow − red earth	[140]
Pedosphaerae	NP	−1	−1	n.e.		1		black soils	[141]
Chthoniobacter	NPK	1		1	1		*Fritillaria taipanensis*	fine-loamy, mixed, mesic Aridic Haplustalf	[36]
Chthoniobacter	chicken manure		1	1	1		*Brassica oleracea*	–	[131]
Chthoniobacterales	N (ammonium nitrate), PK + sugarcane straw	−1	−1				*Saccharum officinarum*	Haplic Ferralsol	[142]
Chthoniobacteraceae	poultry litter + mineral P			n.e.			*Zea mays*	dark-red acid distrophic latosol	[143]
Xiphinematobacter	PKSNaMg + organic/inorganic N	1	−1				*Triticum aestivum*	–	[137]
Spartobacteria	N (urea), P		−1	n.e.		1		black soils	[141]
Spartobacteria	NPKS		n.e.	n.e.	n.e.		cereals (rotation)	Orthic Gray Luvisol (Typic Cryobralf) of the Breton loam series	[29]
Spartobacteria	–	−1	−1			−1	grasses	red–yellow podzolic latosol (Kandiudult with sandy loam texture	[144]
Spartobacteria	N (urea) + mineral PK		n.e.	n.e.	n.e.	n.e.	*Saccharum L*. spp.	–	[145]
Spartobacteria	N (urea), PK + manure	n.e.	1	1	n.e.	−1	*Glycine max*	Chinese Mollisols	[146]
Verrucomicrobiota	NPK and NPK + manure		−1				*Oryza sativa*		[147]
Verrucomicrobiota	manure and straw fertilisation		1				*Triticum aestivum*	non-calcareous fluro-acquic	[148]
Verrucomicrobiota	–	−1	−1	−1	−1	−1	*Halocnemum strobilaceum*,* Phragmites communis*,* Halostachys capsica*	saline-alkaline	[149]
Verrucomicrobiota	NPKCaMg/organic fertiliser		1	1	1		*Euryale ferox*	sandy loam	[150]
Verrucomicrobiota	NPK + cattle manure	−1	−1			1	*Zea mays*	fluvo-aquic and light loam	[151]
Verrucomicrobiota	NPK fertiliser		−1	n.e.	1		*Oryza sativa*	sandy loam	[152]
Verrucomicrobiota	chemical fertiliser		n.e.	−1	n.e.	n.e.	different crops (continuous)	sandy and clay	[153]
Verrucomicrobiota	NPK	n.e.	1	n.e.	−1	n.e.	grasses	heavy-clay	[154]
Verrucomicrobiota	mineral N/straw/manure		−1			1			[26]
Verrucomicrobiota	vermicompost/mushroom residue	n.e.	n.e.	n.e.	n.e.		grassland (mostly *Leymus chinensis*)	chestnut soil	[20]
Verrucomicrobiota									[155]
Verrucomicrobiota	chemical N fertiliser/organic manure	−1	−1	−1			*Zea mays*	Hapli-Udic Cambisol	[156]
Verrucomicrobiota	NPK	−1					*Zea mays*,* Glycine* sp.	Light chernozem	[157]
Verrucomicrobiota		1				1	*Arachis hypogaea*	Hapli-Udic Cambisol	[158]
Verrucomicrobiota	NPK	n.e.			n.e.	1	*Allium tuberosum*	fluvisol	[159]
Verrucomicrobiota	maize straw	1				n.e.	*Zea mays*	Argosols (sandy loam)	[106]
Verrucomicrobiota	NPK + straw	1		−1			*Triticum aestivum/Oryza sativa*	–	[160]
Verrucomicrobiota	organic fertiliser + microbial fungicide	1					*Zea mays*	brown soil with sandy loam and alluvial parent material	[113]
Verrucomicrobiota	biogas slurry + NPKB	n.e.	n.e.	n.e.	n.e.		*Oryza sativa/Brassica napus*	silt loam	[161]
Verrucomicrobiota		1					*Beta vulgaris*	Eutric Cambisol	[21]
Verrucomicrobiota		1					*Oryza sativa*		[162]
Verrucomicrobiota	superphosphate	1		1			*Triticum aestivum*	Eum-Orthic Anthrosol	[163]
Verrucomicrobiota	NPK			−1			Oryza sativa	paddy soil	[164]
Verrucomicrobiota	NPK + sugarcane straw			1			*Saccharum officinarum*	Eutric Cambisol	[165]
Verrucomicrobiota		1			−1		*Mimosa debilis/Senna alata*	Hortic Anthrosol	[166]
Verrucomicrobiota	grass mulching	−1					*Ziziphus jujuba*	torrifluent	[167]
Verrucomicrobiota	N (urea)		−1				*Camellia sinensis*	ultisol, loamy clay	[168]
Verrucomicrobiota	maize straw + NPK	n.e.	n.e.				Crop rotation: *Triticum aestivum/Zea mays*	aquic inceptisol	[151]
Verrucomicrobiota	NPK + cattle manure/poultry manure/urea	n.e.	n.e.			n.e.	*Oryza sativa*	ultisol	[105]
Verrucomicrobiota	bio-fertiliser + rotten maize straw		n.e.	n.e.	n.e.	n.e.	*Avena sativa*	saline-alkaline soil	[169]
Verrucomicrobiota	digestate/liquid urea ammonium nitrate		n.e.			n.e.	*Lolium rigidum*	yellow sandy duplex (typic palexerult)	[170]
Verrucomicrobiota	mineral NPK and organic fertilisers	n.e.		n.e.		n.e.	*Zea mays*	calcaric cambisol	[171]
Verrucomicrobiota	N (urea)		n.e.				crop rotation: *Zea mays/Triticum aestivum*	fluvo-aquic soil	[172]
Verrucomicrobiota	NPK + cow manure	n.e.	n.e.	n.e.	n.e.	n.e.	legume crops	granular sandy loam	[15]
Verrucomicrobiota	N (urea)		n.e.				*Larix kaempferi*,* L. olgensis*	–	[173]
Verrucomicrobiota	NPK + organic fertiliser	n.e.	n.e.				*Triticum aestivum*	medium clay and cinnamon red vertical structural loess	[174]
Verrucomicrobiota	N (urea)		n.e.			n.e.	grassland (*Bothriochloa ischaemum*,* Stipa przewalskyi*,* Stipa grandis*,* Leymus secalinus*)	calcaric cambisol	[175]
Verrucomicrobiota		−1	−1	−1	−1		*Cucumis sativus*	organic	[176]
Verrucomicrobiota	NPK + manure	n.e.	n.e.	n.e.	n.e.	n.e.	*Glycine max*	Chinese Mollisols	[146]
Verrucomicrobiota	NPK	1		1	1		*Fritillaria taipanensis*	fine-loamy, mixed, mesic Aridic Haplustalf	[36]
Verrucomicrobiota	P + chicken manure	1					*Vicia faba*	sandy soil	[177]
Verrucomicrobiota	Mineral NPSCa		−1	n.e.			alpine tundra (graminoids and forbs)	Inceptosol	[178]

For instance, Cong et al. [106], who studied the effect of straw return on maize growth, observed a positive correlation between the concentrations of soil organic matter (SOC) and dissolved organic carbon (DOC) with the relative abundance of Verrucomicrobiota. The same trend has also been shown in two other field experiments [113, 158]. Conversely, other studies have revealed a negative correlation between carbon concentrations and the relative abundance of Verrucomicrobiota in soil [27, 149, 151, 176]. For instance, Li et al. [151], in an experiment based on long-term manure and mineral fertilisation in a maize field, noted that Verrucomicrobiota were negatively correlated with total organic carbon (TOC), and Duan et al. [134] reported a negative correlation between organic C components (including total, dissolved, and light organic carbon fractions) and Verrucomicrobiota during an organic fertilisation experiment in an apple orchard.

The discrepancies in the aforementioned studies may result from diverse life strategies and ecological roles of Verrucomicrobiota members, leading to varying responses of lower taxa to changes in soil C content. A more consistent pattern can be found among members of the Spartobacteria class and *Luteolibacter* genus (Verrucomicrobiia class). Positive correlations between organic carbon content and *Luteolibacter* have been observed in several studies involving both organic and inorganic fertilisation [135–137], as shown in Table 2. In contrast, *Ca*. Udaeobacter (formerly DA101 clade; Spartobacteria class) has been shown to be negatively correlated with soil carbon content [119]. Rao et al. [179] also reported that members of Spartobacteria are more likely to be associated with low soil carbon content. Furthermore, representatives of the Chthoniobacteraceae family, also within Spartobacteria, were significantly more numerous in soils without glucose compared to those amended with sugar [180].

However, even investigations at lower taxonomic levels are sometimes inconclusive. Such is the case of Opitutia, one of the most abundant soil classes. Some studies show a positive correlation between their relative abundance and carbon content [119, 134], while others indicate a negative one [127]. For instance, a study conducted on a mountain slope revealed that the relative abundance of Opitutia rises as the soil C content increases [119]. Similarly, Zhang et al. [181] observed a positive correlation between the genus *Lacunisphaera* (Opitutia) and C content in a chamber experiment where C and N were introduced into apple orchard soil. However, Duan et al. [134] reported opposite patterns in apple orchards amended with an organic fertiliser, detecting a negative correlation between Opitutia and carbon content.

In summary, Verrucomicrobiota appear to be diverse in their capacity for carbon compound transformations, and their variable correlations with organic carbon suggest classification into different life-history strategies, which will be discussed in more detail in another chapter.

###  Nitrogen Cycling

Members of Verrucomicrobiota take part in various steps of the nitrogen cycle such as denitrification and decomposition of nitrogen compounds (e.g. urea) [37, 175]. For instance, soil bacteria *Opitutus* and *Pedosphaera* are regarded as typical denitrifiers, while *Chthoniobacter flavus* is known to perform both denitrification and anammox processes [182, 183]. Bünger et al. [37] showed that both rhizospheric and endophytic bacteria associated with rice may be involved in different steps of denitrification [37]. Numerous members of the class Opitutia are diazotrophs, which mostly applies to symbiotic microorganisms living in the gastrointestinal tract of termites (*Geminisphaera colitermitum*, *T.mucosus*) [42, 182]. However, the same ability is shared by *R. amylovorans* – a strain isolated from a greenhouse fermenter [43]. A *nif* gene has also been found in two rhizospheric isolates, and in a bacterium of the order Opitutales, extracted from a fractured shale [37, 44]. In contrast, *Lacunisphaera* sp., isolated from a constructed wetland, harbours an *nrf* gene, which suggests its involvement in ammonification [184]. Both processes are crucial for plant growth and development as they increase the available nitrogen (AN) pool in soil.

Overall, the ecological role of Verrucomicrobiota in nitrogen turnover highlights their potential sensitivity to changes in nitrogen availability caused by agricultural practices. Since these bacteria participate in both nitrogen fixation and mineralisation, shifts in external nitrogen inputs can substantially alter their community structure and function. Due to the relatively high functional diversity (based on current knowledge) within this phylum, the relative abundance of soil Verrucomicrobiota members responds differently to various types of mineral fertilisation, as illustrated in Table 2. It can be stated with high probability that when nitrogen is supplied as urea, the relative abundance of ureolytic members of Verrucomicrobiota tends to increase, whereas ammonium nitrate application promotes the growth of microorganisms capable of utilising this particular nitrogen source [172, 185]. In contrast, diazotrophic populations may decline under conditions of high mineral nitrogen availability, as external nitrogen inputs suppress their biological nitrogen fixation activity [152, 186, 187].

Therefore, examining how Verrucomicrobiota respond to various N fertilisation is crucial for understanding their ecological adaptability and contribution to soil nutrient cycling in agricultural lands. The following section summarises the current state of knowledge on this topic.

While analysing the impact of N fertilisation on soil microbial community it is necessary to distinguish between mineral and organic amendments, as their effects may differ significantly. Referring to the meta-analysis of Dang et al. [26], there are several studies illustrating how mineral N fertilisation can decrease the relative abundance of Verrucomicrobiota. For example, Qian et al. [139] found that reduced mineral N fertilisation (urea) led to an increase in relative abundance of Verrucomicrobiota in sandy loam soil from rice fields in Jiangsu Province (China). Moreover, the authors reported a negative correlation between soil ammonium content and Verrucomicrobiota [139]. Similar patterns were reported in other studies [137, 178, 183], as shown in Table 2.

However, there are also studies indicating that mineral nitrogen fertilisation enhances the relative abundance of Verrucomicrobiota. For instance, nitrogen application caused an increase in Verrucomicrobiota in the noncalcareous fluvo-aquic soils from maize fields in Shandong Province (China) [129].

Slightly different results, although still diversified, have been obtained in experiments with organic N amendments. As demonstrated in the meta-analysis by Dang et al. [26], the application of organic nitrogen fertilisers, which also provide carbon sources (e.g., cellulose and hemicellulose found in straw or compost), including manure and straw additions, significantly increases the relative abundance of Verrucomicrobiota. Such results were noted in several studies [131, 148, 165, 177], as shown in Table 2. For instance, Chen et al. [148] reported that manure and straw fertilisation increased the relative abundance of Verrucomicrobiota in non-calcareous fluro-acquic soil in a wheat field of the North China Plain. Such results may indicate a proliferation of Verrucomicrobiota taxa possessing genes involved in the degradation of complex biopolymers such as cellulose, hemicellulose, and lignin.

However, there are also studies indicating a decline in the relative abundance of Verrucomicrobiota after the introduction of manure, straw, or other organic fertilisers [147, 151, 188]. For example, manure fertilisation led to a reduction in the relative abundance of Verrucomicrobiota in fluvo-aquic and light loam soils from maize fields in Shandong Province (China). In this case, relative abundance of Verrucomicrobiota was negatively correlated with total nitrogen content [151].

In conclusion, although many studies suggest that the relative abundance of Verrucomicrobiota in soil tends to decline after mineral N fertilisation and report a negative correlation between N content and the relative abundance of phylum [26, 151, 152], this relationship remains inconclusive as some studies show the opposite trend [150]. Such a phenomenon is related to the significant heterogeneity of this bacterial phylum and the varying responses of individual taxa to different types of fertilisation. However, a more detailed analysis of lower taxa within Verrucomicrobiota also does not provide entirely clear answers about the ecological role of individual classes or orders. Most studies show that lower relative abundance of Verrucomicrobiota members is associated with lower soil N content [119, 141]. For instance, Shen et al. [119] demonstrated that Optitutia, Methylacidiphilae, and members of the genus *Pedosphaera* were negatively linked with soil N content along an elevation gradient on Changbai Mountain (China). Interestingly, a similar negative correlation for the genus *Opitutus* was also observed following the application of substantial amounts of urea. This trend may hypothetically be associated with a decline in diazotrophic taxa [22], which are present within the class that includes *Opitutus* [37, 42, 43, 182]. Additionally, a negative correlation between taxa belonging to the class Pedosphaerae, including the order Pedosphaerales, the family Pedosphaeraceae, and the genus *Pedosphaera*, and soil nitrogen content was also reported in other studies [107, 139, 141] (Table 2). Similarly, Zhou et al. [141] found that members of the class Spartobacteria are negatively correlated with both ammonium and nitrate forms of N in a thirty-year long wheat experiment in Harbin (China). Additionally, Kavamura et al. [137] noted higher relative abundances of *Ca.* Xiphinematobacter (Spartobacteria), *Luteolibacter* (Verrucomicrobiia), and *Prosthecobacter* (Verrucomicrobiia) in N-unfertilised soils compared to fertilised soils in wheat fields at Rothamsted Research (UK). Nonetheless, some studies suggest the opposite. For example, Hester et al. [189] found that the Opitutales order was associated with higher amounts of N in a controlled experiment. Likewise, Shen et al. [119] observed that the relative abundance of Spartobacteria responds positively to applications of N.

In summary, these contrasting results highlight the considerable variability in how Verrucomicrobiota respond to nitrogen availability, and likely also to different types of fertilisers. At present, it can be hypothesised that taxa within the class Pedosphaerae generally respond negatively to nitrogen addition in soils.

However, these aspects require further investigation, which should soon be feasible thanks to advances in sequencing techniques and bioinformatic analyses. In particular, we need detailed data on how the relative abundance of Verrucomicrobiota carrying genes such as *ure* (urea hydrolysis), *amoA* (ammonification), and *nif* (atmospheric nitrogen fixation) responds to different nitrogen fertilisers.

### Phosphorus Cycling

Some members of the phylum Verrucomicrobiota are involved in inducing phosphate hydrolysis. They produce enzymes such as alkaline phosphatase, acid phosphatase, and naphthol-AS-BI-phosphohydrolase, which help solubilise phosphate salts [45, 46]. All three compounds have been found in *L. luteus* [190]. Bünger et al. [37], who isolated four strains from rice roots and rhizosphere, found that these isolates not only produce acid phosphatase, but also possess genes for citrate synthase. Wang et al. [191] reported a significant correlation between available P and the relative abundance of *Lacunisphaera* sp. in the cotton rhizosphere. Moreover, this bacterium has been shown to harbour a *gcd g*ene encoding glucose dehydrogenase – an enzyme that induces production of gluconic acid from glucose, thus contributing to phosphate solubilisation. Castillo Villamizar et al. [192] discovered that a purple acid phytase gene ***pho18*** probably originates from bacteria belonging to the genus *Terrimicrobium*, while Garaycochea et al. [193] identified genes encoding acid phosphatase in *Pedosphaera* sp. Given that different Verrucomicrobiota taxa are involved in phosphorus transformations through various mechanisms - such as phosphate hydrolysis and organic acid production – their responses to phosphorus addition are likely to be diverse.

Several metataxonomic studies suggest that Verrucomicrobiota are more commonly associated with low P levels in the soil [149, 164, 178]. For instance, Samaddar et al. [164], who conducted an experiment in a rice paddy field in South Korea, noted a negative correlation between Verrucomicrobiota and available phosphorus (AP) content in soil. Similarly, in the study concerning N fertilisation in alpine tundra soil [178], a negative relationship between Verrucomicrobiota and P content was reported. Zhao et al. [160, 194] observed the same trends in a study examining the effect of straw on the soil bacterial community within a rice-wheat farming system, where the relative abundance of Verrucomicrobiota was negatively correlated with total phosphorus (TP) content [160, 194]. Nevertheless, some studies report positive correlations, suggesting that Verrucomicrobiota responses to P may be influenced by the diversity of taxa involved. For example, in sugarcane fields, Verrucomicrobiota were strongly associated with four forms of P, including organic and inorganic P, both insoluble and retained fractions [165]. Similarly, positive correlations with AP were observed in tomato fields on coastal saline soils in China [47]. All things considered, there is still insufficient data to draw definitive conclusions about the relationship between P and Verrucomicrobiota at lower taxonomic levels (Table 2). The same seems to apply to potassium, as shown in Table 2; the contrasting results indicate that Verrucomicrobiota responses to K availability also vary depending on taxa.

### Soil pH

Soil microbial communities include both acidophilic and alkaliphilic taxa, which show optimal growth under low or high pH conditions, respectively. Agriculture and climate change lead to alterations in soil pH, which plays a key role in shaping the activity and composition of soil bacterial communities [157, 195]. Soil pH influences the physico-chemical properties of the rhizosphere, the structure of the soil, and the ion solubility and mobility. Recent studies show that pH has a greater influence on the relative abundance and distribution of bacterial phyla than nutrient availability [157, 196]. Bacteria differ in their tolerance to pH levels: for instance, Actinobacteria and Firmicutes prefer alkali environments, while the relative abundance of Acidobacteria and Gemmatimonadetes rises as pH decreases [157, 196]. The relationship between soil pH and Verrucomicrobiota, however, is less clear. Some authors report a positive correlation between soil pH and the relative abundance of Verrucomicrobiota [26, 107, 150, 158, 159], while others suggest that members of this phylum prefer more acidic habitats [144, 149, 197]. Shen et al. [119] observed that the response of Verrucomicrobiota to soil pH varies depending on the studied taxon. For example, Opitutia and Methylacidiphilae were negatively correlated with pH, whereas Verrucomicrobiia and Spartobacteria showed a positive correlation with this parameter. However, *Chthoniobacte*r and *Candidatus* Xiphinematobacter (both belonging to the Spartobacteria class) were less abundant in higher pH soils. Moreover, Shen et al. [119] concluded that, at the phylum level, Verrucomicrobiota as a whole showed no clear correlation with soil pH. In conclusion, no consistent trend in response to pH value can be observed at the phylum level for Verrucomicrobiota, and further research is needed to clarify this complex relationship.

## Response to Pesticides

In modern agriculture, pesticides are crucial to maintain high food demand, and large quantities of chemical substances enter the soil environment. Yet, there are no studies that directly investigate the effect of pesticides on Verrucomicrobiota. Available information indicates that soil treatment with the most widely used herbicide – glyphosate, resulted in a slight increase in the relative abundance of Verrucomicrobiota. However, it was suggested that representatives of this phylum may provide a rather supportive, not essential role in the biodegradation of the pesticide [198–200]. Langarica-Fuentes et al. [201] reported significant log2 fold change (1.97 and 2.14) in the relative abundance of Verrucomicrobiia class after a 7-day soil microcosm experiment with 15 mg kg^− 1^ of glyphosate dose. Exposure to other pesticides such as: pyroxasulfone, imidacloprid, thiamethoxam, difenoconazole, atrazine and clothianidin resulted in a decrease in the relative abundance of the Verrucomicrobiota in soils [139, 191, 202–205]. Unfortunately, due to the lack of more in-depth analysis or further studies, it is not possible to say whether this effect should be attributed to the toxicity of the pesticide towards Verrucomicrobiota or rather an increase in the number of other, most likely copiotrophic microorganisms (pesticides are biodegradable and may be used as a source of biogenic compounds).

In most cases, the information regarding the response of this phylum to the above-mentioned pesticides is limited to a few sentences indicating a shift in relative abundance, without further explanation. Due to the vast relative abundance of Verrucomicrobiota in agricultural environments and their potential in nutrient cycling and promoting plant growth, the effect of pesticides, especially commonly used herbicides (i.e. glyphosate) should be further investigated.

## Life-History Strategies of Verrucomicrobiota

The classical r/K life-history framework distinguishes organisms based on their reproductive and growth strategies. Typical r-strategists are opportunistic, rapidly exploiting abundant resources, and usually correspond to copiotrophic organisms, which grow quickly in nutrient-rich environments. K-strategists, in contrast, are conservative, adapted to stable but resource-limited conditions, and are often oligotrophic, efficiently utilising low concentrations of nutrients. However, it is important to note that in many original scientific papers, Verrucomicrobiota and other taxa are frequently explicitly classified as oligotrophic, which may contradict the current state of knowledge. These misclassifications are often repeated in subsequent studies, perpetuating inaccuracies in the literature. For instance, Verrucomicrobiota have been classified as oligotrophs in several recent papers [206–208], however, as mentioned in earlier section, many other studies have reported positive correlations between Verrucomicrobiota abundance and soil nutrient content, suggesting potential copiotrophic tendencies [21, 27, 106, 107, 113, 158, 160, 162, 209]. Such inconsistencies highlight the need for caution when assigning Verrucomicrobiota to strict r/K categories. Analysis of lower Verrucomicrobiota taxa regarding life strategies indicates that many classes and genera exhibit both copiotrophic and oligotrophic traits. As mentioned earlier, the Spartobacteria class, including *Ca*. Udaeobacter and members of the Chthoniobacteraceae family appears largely oligotrophic, preferring soils with low carbon and nitrogen content. Conversely, the Opitutia class shows more variable responses, with studies reporting both positive and negative correlations of their relative abundance with soil carbon content. This variability, typical of highly diverse taxa and compounded by contradictory literature reports, emphasises the need to consider the classification of Verrucomicrobiota according to r/K strategies indicative rather than absolute.

To address these limitations, Ho et al. [111] proposed applying the Competitor–Stress-tolerator–Ruderal (C-S-R) life strategy framework to microbial communities. In this context, Competitors (C) are organisms that thrive in environments with high nutrient availability, efficiently exploiting carbon and nitrogen resources and maximising growth. Stress-tolerators (S) are adapted to environments with low nutrient availability or other environmental stresses, maintaining metabolic activity under limited resources and extreme conditions. Ruderals (R) are opportunistic organisms that rapidly respond to transient nutrient inputs or disturbances, proliferating quickly when conditions temporarily improve [111, 210, 211].

For instance, methanotrophic Verrucomicrobiota can be interpreted within this expanded framework. Representatives such as Methylacidiphilaceae are acidophilic and thermophilic, capable of autotrophic growth using geologically derived methane under extreme pH and temperature conditions. These traits suggest that they function primarily as Stress-tolerators (S), as they are adapted to survive in low-energy, high-stress environments such as volcanic soils. In contrast, methanotrophs inhabiting more nutrient-rich or disturbed environments may exhibit features of Competitors (C) or Ruderals (R), depending on resource availability and environmental stability. Therefore, the C-S-R framework provides a more nuanced classification of microbial life strategies, especially for specialised taxa like methanotrophic Verrucomicrobiota, than the classical r/K model [111, 210, 211].

## Verrucomicrobiota as Plant Growth-Promoting and Biocontrol Bacteria

Plant Growth-Promoting Bacteria (PGPB) are microorganisms that support plant growth through a variety of direct mechanisms, such as the production of phytohormones (including indole-3-acetic acid, IAA), nitrogen fixation via nitrogenase activity, and phosphate solubilisation through the release of organic acids [212]. They can also promote growth indirectly by protecting plants against phytopathogens or abiotic stress, for instance through the activity of 1-aminocyclopropane-1-carboxylate (ACC) deaminase, the production of antibiotic compounds (e.g., polyketides and lipopeptides), or by triggering Induced Systemic Resistance (ISR) in plants [213, 214].

**Fig. 3:**
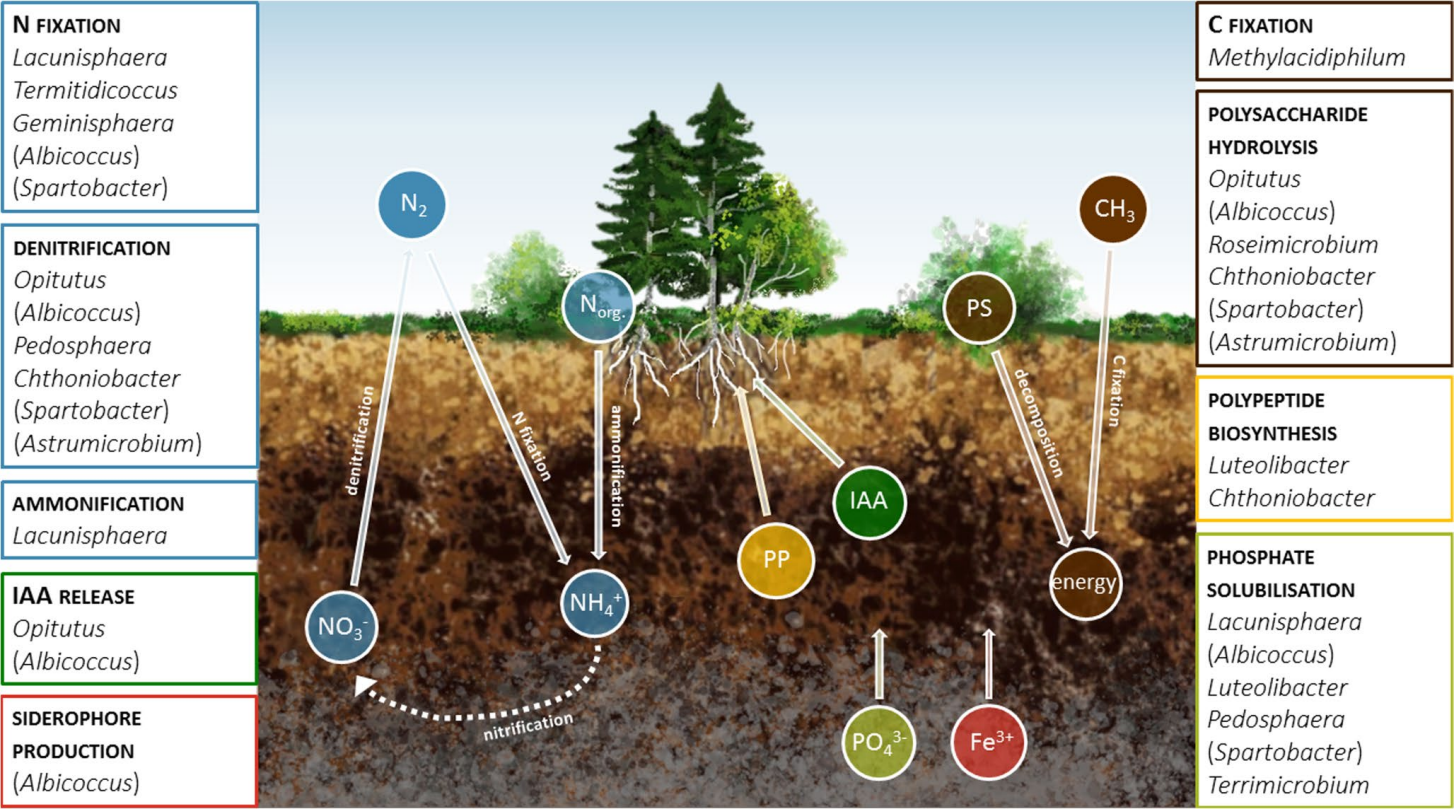
PGP properties of soil Verrucomicrobiota, including their contribution to the C and N cycles; PS - polysaccharides, PP - polypeptides released as secondary metabolites. The names of genera in brackets originate from the publication by Bünger et al. [37] and cannot be found in databases such as NCBI and DSMZ. References: [32, 36, 37, 42, 44–46, 48, 49, 118, 125, 175, 182–184, 190–193, 212–217]

It has been known for some time that members of the Verrucomicrobiota can be rhizosphere-competent bacteria, meaning they are able to move toward plant roots and utilise nutrients from root exudates [33]. Recently, it has been confirmed that Verrucomicrobiota might play an important role in fostering plant growth and development (Fig. 3). One of the key properties of plant growth-promoting rhizobacteria, the ability to produce phytohormone indole-3-acetic acid, has been found in four rice-associated Verrucomicrobiota strains [37]. Furthermore, as previously mentioned, several Verrucomicrobiota strains have *nif* genes, highlighting their potential as directly acting plant growth-promoting bacteria (PGPB) by enhancing nitrogen availability in the rhizosphere [37, 44].

Verrucomicrobiota also demonstrate protective potential against pathogenic organisms, and thus can be considered as indirect plant growth promoters or simply as biocontrol agents. Bacteria from the genus *Chthoniobacter* have been reported to participate in the suppression of plant pathogen *Fusarium oxysporum* in *Arabidopsis* and banana [48, 215]. This activity is probably related to the synthesis of secondary metabolites - an important, but underestimated ability of Verrucomicrobiota [216]. A recent genomic study by Di et al. [217] revealed a great potential of this bacterial phylum to produce such compounds as terpenes, peptides, and polyketides, with *Luteolibacter* displaying the widest range of secondary metabolite biosynthetic gene clusters [217].

Furthermore, as previously mentioned, members of the Verrucomicrobiota are capable of cellulose degradation, which may also facilitate the biocontrol of phytopathogens whose cell walls contain cellulose, such as *Phytophthora infestans*, a member of the fungus-like Oomycota.

Besides, some bacteria within the phylum Verrucomicrobiota exhibit specific traits that make them potentially valuable for bioremediation and plant protection. For instance, genes encoding antioxidant enzymes, such as copper/zinc superoxide dismutase and catalase-peroxidase, have been found in soil bacteria of the genus *Ca.* Udaeobacter [49].

In conclusion, Verrucomicrobiota participate in essential biochemical processes and their activity may affect plant growth and condition. Moreover, in addition to enhanced plant productivity, they can also reduce the negative impact of agriculture on the environment.

## Conclusions and Future Perspectives

Verrucomicrobiota is one of the predominant phyla of bacteria found in soil, and its ecological significance has led to its classification as a keystone phylum. Prior to the advent of modern sequencing techniques, Verrucomicrobiota were widely regarded as an oligotrophic group. However, as our understanding of soil bacterial ecology has deepened through numerous studies, this classification has become debatable. Based on a considerable amount of current evidence, it is now apparent that Verrucomicrobiota include lower-rank taxa belonging to both oligotrophs and copiotrophs, as indicated by their varied responses to soil carbon (C) and nitrogen (N) content. Available literature shows that the class Optitutae comprises both copiotrophs and oligotrophs, whereas the class Spartobacteria predominantly consists of oligotrophs.

The relative abundance of copiotrophs and oligotrophs in the soil can significantly influence the rate of biochemical transformations, such as carbon, nitrogen, and phosphorus cycling. This balance can affect soil health, nutrient availability, and overall ecosystem functioning, making the understanding of microbial populations essential for sustainable soil management. As oligotrophic bacteria are much more difficult to isolate and culture compared to copiotrophic ones, it seems possible that many Verrucomicrobiota species cannot be grown under laboratory conditions [123]. The limitations of bacterial culture may explain the scarcity of studies focusing on soil Verrucomicrobiota, although the relative abundance of this phylum reaches 23% of the total bacterial community [13]. In order to push forward the research it is necessary to develop effective methods of microbial culture. Recently, Tanaka et al. [205] proposed a modification of the “duckweed-microbes co-cultivation method,” which proved useful as a tool to isolate numerous Verrucomicrobiota strains from freshwater samples. This result holds promise for broadening our knowledge about these microorganisms. Development of cultivation techniques may also lead to an increased number of diazotroph detections within the Verrucomicrobiota phylum, enhancing their potential in promoting plant growth. Additionally, as cultivation challenges are overcome, we anticipate an increase in the number of isolates involved in other N cycle-related processes.

Furthermore, the development of third-generation sequencing platforms and improved bioinformatics pipelines is expected to overcome current challenges in studying soil Verrucomicrobiota, particularly those related to their high genetic diversity. Advances in sequencing technologies promise to revolutionise our understanding of Verrucomicrobiota’s role in macroelement cycling and plant growth promotion. It is believed that metagenome-assembled genomes (MAGs) will reveal previously unknown Verrucomicrobiota lineages encoding various carbohydrate-active enzymes (CAZymes), genes coding for enzymes involved in nitrogen and phosphorus cycles, and plant growth-promoting traits beyond what is currently known. However, it should be noted that currently, MAGs from soil environments remain particularly challenging due to the high genetic diversity and relatively low abundance of many lower taxonomic rank Verrucomicrobiota members, thus improvements are still needed in long-read sequencing and hybrid assembly approaches to help recover more complete genomes [218–222]. Consequently, for example, it will be possible to better understand the responses of Verrucomicrobiota taxa possessing genes encoding nitrogenase or ammonifying enzymes to different types of nitrogen fertilisation.

Furthermore, integrating single-cell genomics and metatranscriptomics will likely elucidate the active metabolic pathways and environmental interactions of these microbes, highlighting their contributions to nutrient turnover and plant health. Such discoveries could reshape our perspective on Verrucomicrobiota’s ecological roles, underscoring their potential in sustainable agriculture and ecosystem management [218–222].

In addition, rapidly developing approaches such as machine learning and mathematical modeling, which provide instruments for analysing and interpreting large amounts of data, can be used to uncover links between microorganisms and their environmental functions [124]. These technological advancements, when combined with above mentioned strategies, may also help unlock Verrucomicrobiota’s full potential for carbon, nitrogen transformation, and sustainable applications.

Since most research on the impact of mineral and organic fertilisation comes from Asia (Table 2; Fig. 2B), there is a need to conduct more studies in other locations and climatic zones. Moreover, future research should focus not only on diverse soil types from conventional fertilisation systems to better characterise the occurrence of this phylum in the environment, but also on the influence of plant growth-promoting bacteria (PGPB), which are gaining importance, on members of Verrucomicrobiota [188, 223].

In conclusion, further comprehensive studies are needed to fully understand the ecology and functional roles of Verrucomicrobiota at the genus level or lower level. These bacteria are abundant, diverse, and widely distributed, and much of their ecological and biotechnological potential remains to be explored. Recognising the role and trophic status of the Verrucomicrobiota is also important, as there is a significant positive relationship between the ratio of oligotrophs to copiotrophs and the resilience of communities in the soil. This knowledge may be important for planning activities to maintain the homeostasis of soil ecosystems in times of increasing anthropogenic pressure.

## Supplementary Information

Below is the link to the electronic supplementary material.

ESM 1 (DOCX 29.0 KB)

## Data Availability

No datasets were generated or analysed during the current study.
