# Supplementary material for: Advances and Hotspots in Research on Verrucomicrobiota: Focus on Agroecosystems
Source: Microb Ecol. 2025 Nov 22;89(1):1. doi: 10.1007/s00248-025-02657-3 (PMC12714770; doi:10.1007/s00248-025-02657-3)
Supplement: Supplementary file 1 — (DOCX 29.0 KB) [file 248_2025_2657_MOESM1_ESM.docx]

**Advances and hotspots in research on Verrucomicrobiota: focus on agroecosystems**

**Aleksandra Naziębło^1*^, Anna Pytlak^2^, Adam Furtak^2^, Jakub Dobrzyński^1^***

1 - Institute of Technology and Life Sciences – State Research Institute, Raszyn, Poland

2 - Institute of Agrophysics, Polish Academy of Sciences, Lublin, Poland

*****Corresponding authors: [a.nazieblo@itp.edu.pl](mailto:a.nazieblo@itp.edu.pl); [j.dobrzynski@itp.edu.pl](mailto:j.dobrzynski@itp.edu.pl)

ORCID numbers:

AN: 0000-0002-3536-8804

AP: 0000-0001-7941-1766

AF: 0000-0002-9424-3147

JD: 0000-0002-5816-2745

**Review methods**

The literature survey is based on Google Scholar, Scopus, and Web of Science databases. A number of keywords and their combinations were used to search for publications – for instance “Verrucomicrobiota”, “microbial community”, “bacteria”, “soil”, etc. Articles cited in the reference lists of each publication were also examined. Finally, over 180 peer-reviewed research articles were selected (a more detailed description can be found in the Supplement). The figures were prepared using VOSviewer, Krita, Paint and MS Office.

The systematic review takes into account work produced since 1997, in which Verrucomicrobiota was described as a separate division/phylum based on the unique sequences of the 16S rRNA gene. The search terms took into account changes in nomenclature over the years, which enabled us to obtain a complete picture of the development of directions in Verrucomicrobiota-focused research [1, 2, 3]

Bibliometric data were retrieved from Web of Science Core Collection database on February 3, 2025, using the following term query: (Verrucomicrobia OR Verrucomicrobiota OR Verrucomicrobaeota (All Fields)). The resulting record contained 3,132 publications, including 3,017 articles, 86 reviews, 20 book chapters. Co-occurrence of terms (including title, abstract and keywords) was visualised using VOSviewer (version 1.6.16) with binary counting and the minimum number of occurrences of the term set to “2”. A VOSviewer thesaurus file (thesaurus_terms.txt) was used to integrate synonyms and remove irrelevant terms [4]. The final dataset consisted of 183 terms, which were used for network and overlay visualisation analysis.

References:

1. Arnds J, Knittel K, Buck U, Winkel M, Amann (2010) Development of a 16S rRNA-targeted probe set for Verrucomicrobia and its application for fluorescence in situ hybridization in a humic lake. Syst Appl Microbiol 33:139–148. https://doi.org/10.1016/j.syapm.2009.12.005

2. Orellana LH, Francis TB, Ferraro M, Hehemann J-J, Fuchs BM, Amann RI (2022) Verrucomicrobiota are specialist consumers of sulfated methyl pentoses during diatom blooms. ISME J 16:630–641. https://doi.org/10.1038/s41396-021-01105-7

3. Awala SI, Gwak J-H, Kim Y, et al (2024) Nitrous oxide respiration in acidophilic methanotrophs. Nat Commun 15:4226. <https://doi.org/10.1038/s41467-024-48161-z>

4. Van Eck NJ, Waltman L (2010) Software survey: VOSviewer, a computer program for bibliometric mapping. Scientometrics 84:523–538. https://doi.org/10.1007/s11192-009-0146-3
